# Building a pathway to One Health surveillance and response in Asian countries

**DOI:** 10.1016/j.soh.2024.100067

**Published:** 2024-04-02

**Authors:** Roger Morris, Shiyong Wang

**Affiliations:** a Massey University EpiCentre and EpiSoft International Ltd, 76/100 Titoki Street, Masterton 5810, New Zealand; b Health, Nutrition and Population, World Bank Group, Washington, DC, USA

**Keywords:** Surveillance, Economics, Emerging disease, Genomics, Artificial intelligence, Priority setting, One Health

## Abstract

To detect and respond to emerging diseases more effectively, an integrated surveillance strategy needs to be applied to both human and animal health. Current programs in Asian countries operate separately for the two sectors and are principally concerned with detection of events that represent a short-term disease threat. It is not realistic to either invest only in efforts to detect emerging diseases, or to rely solely on event-based surveillance. A comprehensive strategy is needed, concurrently investigating and managing endemic zoonoses, studying evolving diseases which change their character and importance due to influences such as demographic and climatic change, and enhancing understanding of factors which are likely to influence the emergence of new pathogens. This requires utilisation of additional investigation tools that have become available in recent years but are not yet being used to full effect. As yet there is no fully formed blueprint that can be applied in Asian countries. Hence a three-step pathway is proposed to move towards the goal of comprehensive One Health disease surveillance and response.

## 1 Disease emergence

In brief, a disease is considered to emerge when it occurs in a new species not known to be susceptible to a previously known or unknown pathogen, when it occurs in more severe form (substantially higher incidence and/or more severe effects on hosts), or when a genetic variant may behave in unexpected ways in host species already known to be susceptible to the pathogen. Recent examples of the first type of emergence include SARS-CoV-1 and SARS-CoV-2 in people. Examples of the second type include African swine fever in Eurasia and Ebola in urban communities of West Africa. Examples of the third type include avian influenza H5N1 directly transferring from wild to domestic birds — and rarely to people — without the involvement of pigs as a step in the transition, and influenza A viruses becoming pandemic in 1918 and on later occasions.

Diseases may also re-emerge after a period of low incidence or severity when circumstances change, such as when previously effective control or treatment methods are no longer as effective. Malaria and tuberculosis have re-emerged in various countries in this situation. Rinderpest in cattle re-emerged in Africa after being considered close to eradication, but was later eradicated globally by a modified control program. Changes in land use patterns due to expanding human populations and altered food production systems can precipitate emergence of diseases [1,2], economic pressures may increase human exposure to new pathogens [3] and long-distance movement of wild animals for consumption or other purposes can expose populations to novel pathogens [4,5].

In a study of the 100 largest zoonotic disease outbreaks in comparison with 200 randomly chosen less substantial outbreaks out of a total of 4463 outbreaks [6], it was concluded that large outbreaks had more epidemiological drivers contributing to their development, particularly changes in vector abundance, human population density, unusual weather conditions and water contamination. Pathogens causing large outbreaks were more likely to be viral and vector-borne.

Evidence has been put forward that bats and rodents are particularly prone to act as the source of emerging diseases. Potential explanations have been provided for this in the case of bats [7,8], showing that they have a higher number of viruses per species than rodents, whereas another study catalogued 5491 rodent-associated viruses [9]. However conversely it has been argued that the importance of bats and rodents is because of the species richness of these taxa [10], not because they carry a disproportionate viral burden. It has also been argued that although bats carry the most virulent zoonotic viruses, the death burden from zoonotic agents is independent of the host species, and more dependent on other factors [11].

Several methods have been used to predict which organisms [12,13], host factors [14,15], environmental factors [16], human demographic factors [6] and other factors may facilitate emergence of new diseases. However multiple drivers are likely to interact to produce future emergence of important new diseases, just as they have with past pandemics. A One Health surveillance system needs to be designed with broad coverage and no predetermined expectations, so that it can detect the unexpected if it occurs. This fundamental assumption forms the basis of the approach proposed in this article.

## 2 Objectives of a One Health surveillance system

Over recent decades there have been numerous examples of the emergence of pathogens from animals to cause establishment and extensive spread in human populations. Consequently there is much greater recognition of the need to integrate human and animal disease surveillance [17]. International organizations have collaborated to formulate a One Health Joint Plan of Action (2022–2026), which provides a framework for converting widely discussed principles into practical action. The step which remains incompletely and unevenly resolved is how to achieve this in individual countries and regions.

The greatest challenge for a One Health strategy is to detect and respond to emerging and re-emerging diseases of animal origin before they pose a risk of causing epidemic or pandemic spread in either human or animal populations. However that objective alone is not sufficient to ensure continued implementation of an expanded surveillance program in Asian countries. A global study of One Health surveillance systems [18] identified 41 systems, including 5 in Asia, but almost all systems were concerned with known disease causal agents, not emerging diseases.

Adding a One Health component to a national or regional surveillance program aims to enhance understanding of the epidemiological behaviour of known and putative zoonotic pathogens within the ecosystems of the region, to assess the impact of changing environmental conditions such as climate change on occurrence and distribution of these pathogens, and to apply additional and improved methods of exploring factors which influence disease processes in the Asian region. A three-step process is proposed to achieve this at country and regional level.

## 3 Step 1 Identify disease risks and set action priorities

The initial step is to conduct a national or regional assessment of the issues which should become the focus of investment in One Health surveillance, and the initial priority which should be assigned to each activity. The procedures to be followed in this assessment are described in the Tripartite Zoonoses Guide [19]. The Guide is supported by a series of operational tools on joint risk assessment [20], surveillance and information management [21] and multisectoral coordination mechanisms [22]. These are further supported by training materials. The provision of effective governance at the human–animal interface is addressed in a separate document [23].

Although procedural steps to formulate a plan to detect and manage zoonotic diseases in a country are well described, implementing them in Asian countries has proved to be challenging [24,25]. A weakness in building a surveillance strategy to achieve those objectives is determining how to go beyond classical event-based surveillance to a more comprehensive investigational approach. This three-step process is designed to fill that gap.

## 4 Step 2 Add tools to enhance a national surveillance system to achieve “One Health” objectives

**Fig. 1:**
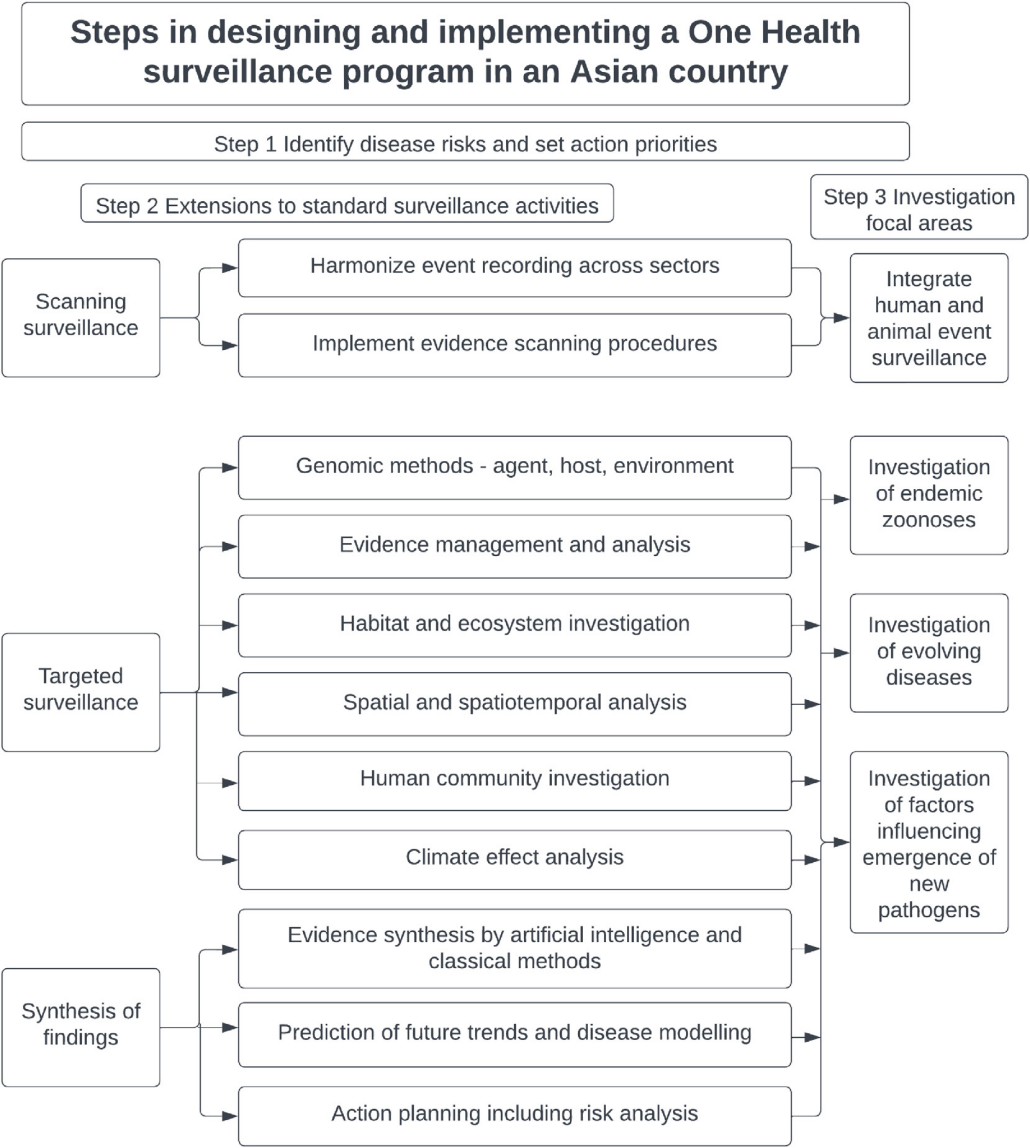
Design of a three-step plan for implementing One Health surveillance in an Asian country.

Existing disease surveillance systems are of value for known diseases present in a country, usually with separate systems for human and animal diseases. These typically use an event-based approach based on recorded disease incidents to measure only numerators, rather than relating numerators to validly measured population denominator data. Therefore current systems in most countries have limited utility for investigating the possible emergence or re-emergence of diseases and pathogens, using a One Health approach. Potential enhancements to existing surveillance tools to provide the expanded functionality will now be reviewed, and a strategy proposed to integrate them into a more effective surveillance program, shown in Fig. 1.

## 5 Genomic tools

Among the new technologies for surveillance [26], genomic technologies offer one of the most important advances. High-throughput sequencing has made it possible to undertake much more comprehensive investigation of samples collected from a wide range of sources, both directly from hosts and from a range of environmental sources. As well as the usual collection of samples from people and animals to identify the presence of known pathogens, more refined investigations can be undertaken. They can identify the range of host species in which an organism of interest occurs, characterise previously unidentified organisms, and link both host and agent specific environments.

### 5.1 Agent investigation

Both DNA and RNA can be used to identify organisms from either hosts or environmental samples [27], so RNA viruses can be investigated as well as DNA organisms of all kinds. Human and animal hosts can be sampled in well understood ways. In addition, samples can also be obtained (for example) from wastewater and other water bodies for viruses [28,29] and for evidence of antibiotic resistance patterns [30], soil [31], directly collected faeces [32,33], animal slurry [34], human cellular debris either deliberately or unintentionally [35] and even mosquito excreta [36].

### 5.2 Host population investigation

Animal populations of substantial size can be investigated using genomic technology to determine the range of known and previously undetected viruses present in the population [37]. A study in pigs found that environmental samples and group samples were more likely to yield positive results by PCR in sampling for influenza A than samples taken from individual animals [38].

Genomic methods have also proved extremely valuable in investigating development of disease outbreaks as they occur [39], and were very influential in managing the COVID-19 pandemic. A growing range of methods in genomic epidemiology are being developed to investigate disease outbreaks [40]. Whole genome sequencing and metagenomics can be used for investigation of outbreaks of foodborne as well as infectious diseases [41]. They can also be used for risk assessment in relation to microbial pathogens [42] and antimicrobial resistance [43].

In principle metagenomic methods can be used to explore the virome associated with host species which may be sources of emerging pathogens [44], but this needs to be undertaken with a pre-defined purpose rather than as a fishing expedition.

### 5.3 Environment investigation

A valuable direction for investigating infectious diseases using genomic methods is to combine ecological approaches in relation to host populations with investigation of the pathogen(s) of interest, including their phylogenetic evolution [27,[45], [46], [47]]. While very useful, this also carries risks related to its validity and repeatability over time [48]. It has even been possible to use capture-recapture sampling of wild birds in conjunction with genomic characterisation to investigate the detailed features of avian influenza infection and transmission [49], and sampling of wild birds has been used to study antibiotic resistance patterns [50]. A study in the Congo assessed the distribution of coronaviruses in a range of wildlife species, and found that almost all of the coronavirus infections were in bats [51].

For epidemiological purposes, it is very valuable to collect samples in a structured way which allows results to be linked to spatial and spatiotemporal information [52,53], including human population movement data [54]. SERAPHIM software can be used to support this [55].

### 5.4 Enhancement of sample processing

In general, samples for DNA and RNA study are collected in the field and transported to laboratories, where efficient and accurate investigations can be implemented [56]. Methods are being developed to improve the sensitivity, specificity and robustness of methods used for analysis of DNA in various sample types [57]. It is becoming increasingly possible to also conduct PCR and genomic sequencing in the field where necessary [58]. It is essential that collection, handling and testing techniques follow appropriate protocols [59]. It is possible to store samples at low temperature for further investigation at later dates [60].

## 6 Epidemiological tools

### 6.1 Information management and analysis

To integrate data from different sectors and where appropriate different countries, it must be possible to harmonise the data from diverse sources. Within a country multiple incompatible systems are commonly used, so it would be desirable to move towards a more integrated approach. Ideally a minimum number of compatible databases would be used to store the data so that appropriate analytical strategies can be used with confidence across the full range of available evidence.

The most widely used information system for low and middle income countries (LMICs) is DHIS2, developed and managed by the University of Oslo (dhis2.org). It operates at national level in 72 LMICs for one or more aspects of health, and is widely used across Asia, with the notable exceptions of China and India, although India has users at State level. DHIS2 has a surveillance and early response component which is used in 42 countries, including seven in Asia. Within the surveillance component it has a sub-component which allows both animal and human data to be added by the relevant government agencies and interpreted jointly. There is also a component dealing specifically with climate-sensitive diseases, and another dealing with integrated surveillance and response. All the components have a range of analytical tools covering most of the needs of countries.

Use of the system has been described for Bangladesh [[61], [62], [63]], Bhutan [64] and Sri Lanka [65,66]. Experience in Bangladesh [63] found the system was of value, but there is a need for significant improvement in its local implementation. A review based on published evidence [67] which included three Asian countries and 13 African countries found that the system was being used for decision-making. The only reported study dealing with the use of DHIS2 in surveillance for epidemic-prone diseases was undertaken in Guinea 2015–2020 [68]. While completeness of submitted reports was 98.5% and timely receipt at national level was 72.2%, the proportion of cases of relevant diseases which were reported was lower than desired, and varied between diseases. Important benefits of standardizing the software tools used for surveillance across LMICs include avoiding the development of a plethora of different dashboards to access and analyse data in an outbreak situation [69], and increasing comparability of data between countries.

Activities that make it easier to submit information and therefore make case-based reporting more effective include use of mobile phones which are becoming almost universally available in many countries [[70], [71], [72]]. A review of 110 studies of electronic surveillance methods [73] found that only 25% were applied in LMICs, and only three dealt with outbreak investigation. Evidence derived from internet use through Google community mobility patterns has been used to show differences in population mobility between countries during the COVID pandemic [74], and has been used in India [75] for this purpose. However it is difficult to minimise bias within and between LMICs resulting from unmeasured differences in internet access between populations. Bias is much less of a problem when data are collected electronically by remote sensing and other indirect methods [76], but interpretation and utilisation of the large quantities of data available by these methods requires specialist skills. Artificial intelligence (AI) tools provide one of the solutions to this problem [[76], [77], [78]], as discussed below. By collecting a library of past epidemics, it becomes possible to improve prediction of the behaviour of new epidemics [79]. Point of care testing using biosensors [80] can provide a way of obtaining disease surveillance data from a much wider range of locations than traditional testing in laboratories.

### 6.2 Habitat and ecosystem investigation

An important challenge in developing surveillance systems for emerging diseases is the uncertainty about which pathogens might emerge, and how they might transfer from the ecosystem in which they naturally occur to establish and maintain infection in the human population. There are epidemiological approaches which can provide valuable insights into the emergence of a pathogen which has potential to cause a human epidemic.

Habitat investigations can develop much deeper understanding of an inadequately understood pathogen, while also building national expertise in investigation methods. An Asian example of investigating an emerging disease is a series of linked investigations of the disease scrub typhus, caused by *Orientia tsutsugamushi* and transmitted by trombiculid mites of the genus *Leptorombidium,* which has emerged in Bhutan and nearby countries in recent years. This was first recognised when an increase in cases of pyrexia of unknown origin was observed. The disease is spread by rodents associated with human habitation, and hence is synanthropic in its epidemiology. Following initial identification of the organism in Bhutan in 2008 [81], and availability of a test for the organism in 2014, a retrospective study was conducted across hospitals to determine the clinical and epidemiological pattern of the human disease [82]. Then a case control study was undertaken [83] to identify risk factors, which showed that harvesting cardamom (*OR* = 1519.0) and living in traditional housing (*OR* = 472.3) were the most important risk factors in a conditional multivariable analysis. A linked study of rodents and their associated pathogens using epidemiological and phylogenetic methods identified four additional zoonotic pathogens not previously known to be present in Bhutan [84]. Cardamom farming is a recently developed business in Bhutan, and the process of clearing land and then harvesting cardamom has been an important cause of increased rodent-derived zoonotic infections. Practices are therefore being developed to reduce the exposure of people in the high risk southern districts of Bhutan [85].

Among the factors that increase the risk of transfer of pathogens to people is involvement with animals [86], and especially with wildlife used as food [87]. This can occur in daily activities [88] for rural populations, but also for a wider population through consumer markets [87,89] and commercial trade [90,91]. Increased exposure to wildlife species and their pathogens has come in part from changes in land use as populations have grown [92,93], and from fragmentation of land use resulting in greater exposure to pathogens [94]. In areas where forests are surrounded by areas of high density of people in poverty, this is likely to precipitate emergence of more diseases [2], including in Nepal and Bangladesh. In India multiple ecological factors and human practices have contributed to the impact of zoonoses [95].

Ecological niche modelling and species distribution modelling [96] can be used to investigate and predict the location in which particular hosts or pathogens may be found [97,98] or inferred to be present [[99], [100], [101]]. Evaluations have been conducted for Asia of the spatial variation in risk of spillover of SARS-related coronaviruses from bats to people, using various epidemiological factors [102,103]. Another study evaluated global bat diversity and proposed that emergence of the SARS viruses was possibly related to climate change [104]. A study of the bat-transmitted Hendra virus in Australia suggested that climatic changes would shift the locations at which Hendra infection occurred [105].

At an ecosystem level, it is possible to explore the influence of various factors on emergence of diseases. Avian influenza is a major hazard causing disease in domestic poultry, and in people in a form that is of low contagiousness, but has the potential to become substantially more contagious — with pandemic potential. Infection is maintained at regional and global scale in waterbirds and transmitted to other species [106], so understanding the way in which this occurs in various ecosystems is an important part of emerging disease surveillance in Asia.

### 6.3 Spatial and spatiotemporal analysis

Spatial and spatiotemporal analysis can provide valuable insights into the geographical distribution of diseases, pathogens and hosts at all levels from within hospitals [107] to within-country in Asia [[108], [109], [110], [111]], between country [112,113] and global [96,114]. Analytical methods are available to facilitate these analyses [[115], [116], [117]].

These methods can be used for allocating resources to priority areas for surveillance [[118], [119], [120]], for identifying disease clusters [121,122] or high risk areas [123], for early detection of potential outbreaks [124] and for forecasting future disease occurrence [125,126].

Phylogeography is a particular form of spatial analysis in which genomic data is combined with spatial location data to map the distribution [127] or spread of pathogens [128,129]. Specific tools are available for phylogeographic analyses [[130], [131], [132]]. The method can also be used for epidemiological hypothesis testing [133].

### 6.4 Human community investigation

Methods are available to investigate human populations [134] and joint activities of people and animals in relation to disease transmission and emergence [88,135]. Social network analysis can be used to explore the relationships between people in relation to disease transmission [136], and can be enhanced by combining it with genome sequencing [137]. For One Health purpose, it can be used to investigate animal movement patterns [138] or jointly investigate the movement of people, chickens and materials as predictors of spread patterns of avian influenza [139].

Despite the importance of human movement patterns in disease transmission, it is very challenging to document these patterns, especially in resource-poor environments. Modelling techniques have been applied to represent movement patterns when direct data is unavailable [140,141]. This can be further enhanced by combining it with genomic analysis [54,142]. At the regional scale, range expansion of zoonotic diseases may be influenced by large scale movement of people across national borders due to adverse climatic or security developments [143,144], which must be considered in regional surveillance strategies.

### 6.5 Climate effect analysis

Climate effects are likely to be the single most important influence determining which known diseases grow most in burden on the Asian population over the coming decades. A review of expected changes in the epidemiology of zoonoses [145] concludes that there will be geographical expansion and potentially changes in seasonal pattern of diseases which transmit more effectively at higher temperatures [[146], [147], [148], [149], [150], [151]] or which benefit from changes in vector abundance [[152], [153], [154]] A range of the habitat and ecosystem factors discussed earlier will interact with climatic effects, complicating predictions.

It will be necessary to conduct repeated evaluations of the climate sensitivity of diseases as global and regional changes occur [155]. This will include spatiotemporal analysis and predictive modelling of the interacting effects of climate and other factors [156,157].

## 7 Synthesis tools

The amount of evidence in the form of data, analytical findings, opinions, and other relevant pieces of information on disease epidemiology in Asia is growing extremely rapidly. Better ways of integrating the plethora of evidence into a form that can be used for decision making will be needed. Tools are available to assist with synthesising the evidence into a more coherent form.

### 7.1 Evidence scanning

Epidemiologists now have to draw far more heavily on evidence and rumours [158] from unofficial sources to monitor unexpected events than on official sources, because unofficial sources provide more comprehensive data items and are closer to real time. However separating fact from fiction is challenging and never completely effective. There are now many organizations which provide material that has been moderated to a greater or lesser degree — involving fact checking, cross-checking and other validation procedures — to distill the flood of material into a coherent summary and provide readers with a balanced assessment of available evidence. ProMED Mail has become the global standard for this purpose, covering human, animal and plant health and available without charge. There are numerous other free and subscription concentrators of information in both broad and specialized fields.

### 7.2 Disease modelling and prediction

It is important to use surveillance data to predict the expected occurrence of diseases under various assumptions concerning future trends in climate, human and animal population characteristics, changes in land use and other factors that may be important for particular diseases. A variety of types of epidemiological models are used for evaluating zoonoses, ranging from deterministic spreadsheet models to stochastic simulation models, compartment models and differential equation models. Each has its uses and limitations. Models which incorporate spatio-temporal features [159] and climatic influences [112] allow more targeted evaluation of the effects of diseases in specific locations, and models can be used to design national control policies linked to surveillance [160]. Models that incorporate AI components are increasingly being used, for example in dengue prediction [[161], [162], [163]].

Similarly, a range of economic models can be used to evaluate alternative policies. Integrated modelling systems which cover both epidemiological and economic components in a single analytical system have also been developed [164].

Risk prediction is another valuable synthesis tool [165], used to assess the spatial distribution of infection risk under the influence of changing epidemiological factors [113,123], to evaluate food-borne risks [43] or to assess exposure risk [166]. Genomic analysis [167] may be included.

### 7.3 Artificial intelligence tools

A major challenge in interpreting findings obtained through One Health surveillance is to integrate diverse pieces of information into valid conclusions, which have adequate sensitivity, specificity and repeatability. Traditionally epidemiologists do this using analytical methods to separate out the causal factors of interest from other confounding factors. An alternative approach that is increasingly being used is to apply AI methods that make no initial assumptions about the data, but use the data to learn how to interpret patterns in it, and hence predict the future development of the process of interest. This approach uses much larger volumes of data than classical methods, but can incorporate very diverse data sources of the kind that One Health surveillance makes available. AI tools can be trained using supervised learning to interpret patterns across multiple data streams [168,169] or identify anomalies indicative of an unusual development [170], and can then be used in real time to interpret incoming data and continue to learn from accumulating data as it arrives.

The specific individual methods and software tools are too numerous and diverse to describe here, but they are used across health care [171,172], public health surveillance [173] and animal health [174]. More specialized applications include the use of AI to predict the source host of a newly identified virus [175], using ecological and climatic data to predict West Nile virus outbreaks [176], and managing antimicrobial resistance [177]. A literature review of the use made of AI tools in the COVID-19 pandemic [178] provides a description of techniques and illustrations of how the various methods have been applied.

Various of the tools have already been used in Asia for forecasting dengue [161] and tuberculosis [179], and for extracting content from low-resourced Asian languages [180], while other activities are under development [181,182].

### 7.4 Software tools to facilitate data synthesis and interpretation

A variety of general purpose and specialised software tools are available to assist in the design, management and evaluation of surveillance systems. Most are free and open source, so accessible to all users. A book that covers several aspects of surveillance design and evaluation [183] includes the EVA Survtool, which provides a structured process for planning and evaluating surveillance programs. A decision support tool [184] guides users through a comprehensive structured risk assessment and response design process, and a structured analytical system guides the analysis of outbreak data [185]. A variety of more specialized tools can be used for specific purposes within the surveillance system, and tools can also be developed by project participants using the EpiHacks method [186,187].

## 8 Steps in building the pathway to One Health surveillance

There is considerable global discussion about options for developing One Health surveillance. Bringing together human, domestic animal and wildlife components into a single surveillance strategy is challenging. In addition to endemic diseases and known disease risks, it has to deal with emerging diseases and known diseases which are likely to undergo epidemiological evolution.

### 8.1 Assigning costs and effort in proportion to benefit

Asia is a high risk region for the emergence of diseases which have epidemic and pandemic potential. The region is at risk of adverse climatic changes that will not only directly affect the distribution and epidemiological behaviour of a range of diseases, but are also likely to produce increased movement of people and increased pressure on land use resulting in incursion into areas with substantial wildlife populations.

The benefit from prompt detection and response to emerging and re-emerging infections accrues at global level, whereas the costs and effort required to provide the evidence accrue at national and regional level, and are higher than many Asian countries can afford to pay. The annual benefit of the effort is relatively low for an individual country, because it accrues only when important discoveries occur, but the global benefit if a pandemic is prevented is extremely high. Therefore it is appropriate that the costs of this activity be met mainly from global sources both for currently unknown organisms circulating in wildlife, and for known but incompletely understood organisms such as avian influenza variants and coronaviruses. The organisms of greatest interest in this context are those spread directly between hosts, mainly by the respiratory route.

The benefit from developing improved detection and management strategies for diseases which are likely to expand their range and severity under the influence of changes in climate, human population distribution, land use changes and other ecological considerations will mainly accrue at regional level, because these changes are likely to occur across large parts of Asia. Therefore it is appropriate for countries to collaborate in achieving better understanding of these diseases, and to share the costs. However the benefits of developing improved control methods for these diseases are likely to accrue beyond Asia, so costs could appropriately be shared between global and regional levels. Many of the important diseases in this case are vector-transmitted (dengue, Crimean Congo haemorrhagic fever, Japanese encephalitis) while others tend to be linked to occupational or environmental exposure (leptospirosis, Nipah virus).

Development of enhanced surveillance will also benefit control of endemic diseases in participating countries by providing better evidence on at-risk and affected populations, and helping to direct resources to activities which provide greatest benefit. Evidence arising from the Global Burden of Disease (GBD) program is used extensively by LMICs to allocate health resources. However despite the importance of zoonoses in many of these countries, only five zoonoses and five diseases which have zoonotic components are included among the 369 conditions covered, and data for these diseases is typically very incomplete. Therefore One Health surveillance would give individual countries much better evidence to guide decisions on control of endemic and emerging zoonotic diseases, so it is appropriate that they contribute significant effort to evidence gathering and interpretation.

## 9 Building the pathway for participating Asian countries

### 9.1 Foundations for a One Health surveillance strategy

The strategy will require the participation of several countries in order to mount effective regional surveillance for emerging diseases, with leadership by key people within the countries [25]. It is essential that the issues be seen from the viewpoint of animal owners as well as decision makers, since this is likely to influence their willingness to become involved and to act on information [[188], [189], [190]].

Each country in the Asian region has particular capability in one or more areas of activity described above, and the strategy must build on existing systems in the countries. As part of the initial phase of developing regional surveillance, it would therefore be desirable that centres of excellence in particular aspects of surveillance activity be identified in one or more participating countries. These centres should be resourced to provide training and support on this component of surveillance to other countries in the region, to ensure that all countries are operating at a comparable level of expertise. It will also be necessary for countries to reach agreement on what types of information will be made available beyond national borders, and in what form.

Surveillance can be divided into two categories – scanning and targeted. Scanning surveillance provides an overview of the disease situation from various perspectives, whereas targeted surveillance is designed to provide understanding of specific issues. Both are required for an effective surveillance system. Individual countries may undertake a sub-set of the overall portfolio of surveillance activities needed for effective One Health surveillance.

## 10 Step 3 Undertake expanded surveillance activities on identified focal issues

### 10.1 Scanning surveillance

Event-based surveillance activities need to be integrated between human and animal health.

#### 10.1.1 Harmonise event recording across countries and sectors

Most Asian countries have event recording systems for human health, and several already have systems for surveillance data. Several also have systems for recording event data for animal health. Few have records for wildlife. Experience in working with these countries has shown that units within the national human and animal health systems operate information systems that are incompatible in various ways with systems used in other units, making integration of data difficult or impossible. Because DHIS2 is the most widely used system in LMICs and is open source, it would be ideal to either have all units use DHIS2 with or without modification for local needs, or establish data transfer procedures to back up data from existing systems into DHIS2. This would enable similar analytical methods to be used on data from all participating countries. Some countries are reluctant to share raw data, but summary data could be shared, either with country identification, or collated across countries to produce regional overviews, with variation across the range of participating countries summarised in a form which protects the identity of participating countries.

Event data could then be used to monitor and investigate epidemiological features of the occurrence of different diseases across the group of countries.

#### 10.1.2 Evidence scanning

Evidence scanning is already being undertaken by some Asian countries, but there would be merit in using AI to widen the range of languages which can be searched for relevant information. Rather than building a specific system for use in the region, existing AI-based tools such as Intelliriver Source (https://intelliriver.systems/#home) could be used to collate, manage and distribute information to participating countries.

### 10.2 Targeted surveillance

This requires funds, human and physical resources, and multi-year commitments from both participating governments and donor organizations. There must therefore be identifiable short and medium term benefits beyond the prospect of perhaps identifying an emerging pathogen early in its emergence, important though that objective is. Therefore a triple path strategy is proposed, each of which involves components related to the human and animal populations, both domestic and wildlife. The activities would enhance the capability and capacity of countries to respond to new zoonotic disease challenges as they arise. The tools described above would be used in each of the investigations, where appropriate.

#### 10.2.1 Epidemiological investigation of endemic zoonoses

By undertaking comprehensive epidemiological investigations of carefully selected zoonoses within important habitats in the region, three objectives can be achieved. Methods of investigation beyond those previously used on the disease can be applied, thereby enhancing expertise in these techniques when applied to known organisms and hosts. Secondly, understanding of the selected disease would be enhanced, and control policies could then be re-evaluated. Thirdly, while gathering data on the zoonosis of concern, epidemiological and genomic evidence would be obtained on other hosts, pathogens and unknown organisms in the study area, and could be applied to the other two investigation paths.

#### 10.2.2 Epidemiological investigation of evolving diseases

Numerous diseases are evolving and in some cases extending their range and changing their genetic mix — dengue, Japanese encephalitis, avian influenza and scrub typhus, to name just a few examples, each of which has a different transmission mechanism and ecological influences.

The principal factors driving these evolutionary changes include changing climatic conditions, land use modification, greater interaction with wildlife both in natural habitats and in marketing systems, and shifts in human and domestic animal population density, distribution and movement patterns. These changes are occurring across Asia, and require that diseases of concern be investigated in an ecosystem's framework across the region rather than just within national boundaries. Countries could benefit from joint exploration of these diseases by sharing expertise and knowledge.

#### 10.2.3 Epidemiological investigation of factors influencing emergence of new pathogens

The number of organisms that could emerge as regionally or globally important is far too large to predict with any confidence which ones are at highest risk of causing major problems. Efforts are being made to narrow the list of most likely contenders by various approaches [9,191,192] and to seek to identify the reservoir hosts of these organisms [14,48,175]. An approach which deserves high priority is to enhance understanding of the routes by which a new pandemic organism might emerge [2,193], and the epidemiological factors which could enhance that risk [103]. The next step is to develop ways of reducing the risk and preparing to respond quickly [16,194,195]. Host behavioural factors which can modify risk include movement patterns of animals and people [4,5,144], and types of contacts between people and wild or domestic animals [196,197].

The investigation of these factors and their interaction is a global research priority, and should be supported at global level to provide a framework of understanding that can be used to raise the level of preparedness for a future highly transmissible and pathogenic organism.

## 11 Synthesis of surveillance evidence

A major challenge with mounting an effective surveillance strategy using multiple components in several countries is how to interpret the very large volume of data that will inevitably be collected and which will vary greatly in value and reliability. However analytical and predictive methods have improved greatly in recent decades, as shown in the section on synthesis tools. In combination with more traditional analytical methods these tools can be used to extract meaning from a complex web of data, and in doing so the tools can be refined to further increase their value.

## 12 Dealing with barriers to implementation

Based on experience over several decades of contributing to disease control efforts across Asia, a major achievement within most of the countries has been a substantial growth in the number of trained, enthusiastic and capable people working on disease control programs.

However shifting the behaviour of national organizations and their policies towards a more innovative and facilitating approach has been slow and quite variable between organizations, thereby restricting the extent to which capable people could achieve their full potential on behalf of their countries. This has led many of these people to leave their home countries for places where they could better use their abilities.

The emergence and spread across Asia of novel pathogens such as avian influenza and COVID-19 has been a valuable stimulus to political and organizational recognition of the economic and social consequences of emerging diseases, and the need to be more pro-active in preparing for future disease events. Consequently, more resources are being provided to enhance response capacity, from both national governments and external funders. This is an essential precursor to implementing the approach outlined in this article. The title of the article recognises that there is no neat package of solutions, but rather the opportunity to test out the value of different components of an integrated approach, and to build on experience of individual countries through a cooperative regional approach. The pathway described here needs to be built over time, with leadership from within the region.

## 13 Conclusion

Designing and implementing an effective One Health surveillance strategy in Asia is far from easy, but the range of tools available and their value have both grown rapidly in the last decade. What is needed is to adopt a carefully structured and integrated regional approach, to build on and enhance the existing capability and capacity of the countries. This will require investment of sufficient resources to provide the information needed to prepare for a possible next pandemic, and to reduce the probability that it will be allowed to grow to that scale of event. Resources are now being provided to support the development of such an approach, and this paper outlines a structured approach to achieve this objective.

## Funding

No specific funding was provided for preparation of this article, which has been written as part of the planning for a World Bank project to which both authors are contributing.

## Declaration of competing interest

The authors declare that they have no known competing financial interests or personal relationships that could have appeared to influence the work reported in this paper.
